# Correlates of Total Sedentary Time and Screen Time in 9–11 Year-Old Children around the World: The International Study of Childhood Obesity, Lifestyle and the Environment

**DOI:** 10.1371/journal.pone.0129622

**Published:** 2015-06-11

**Authors:** Allana G. LeBlanc, Peter T. Katzmarzyk, Tiago V. Barreira, Stephanie T. Broyles, Jean-Philippe Chaput, Timothy S. Church, Mikael Fogelholm, Deirdre M. Harrington, Gang Hu, Rebecca Kuriyan, Anura Kurpad, Estelle V. Lambert, Carol Maher, José Maia, Victor Matsudo, Timothy Olds, Vincent Onywera, Olga L. Sarmiento, Martyn Standage, Catrine Tudor-Locke, Pei Zhao, Mark S. Tremblay, ISCOLE Research Group

**Affiliations:** 1 Children’s Hospital of Eastern Ontario Research Institute, Ottawa, Canada; 2 University of Ottawa, Ottawa, Canada; 3 Pennington Biomedical Research Center, Baton Rouge, United States of America; 4 University of Syracuse, Syracuse, United States of America; 5 University of Helsinki, Helsinki, Finland; 6 University of Leicester, Leicester, United Kingdom; 7 St. Johns Research Institute, Bangalore, India; 8 University of Cape Town, Cape Town, South Africa; 9 School of Health Sciences / Sansom Institute, University of South Australia, Adelaide, Australia; 10 Faculdade de Desporto, University of Porto, Porto, Portugal; 11 Centro de Estudos do Laboratório de Aptidão Física de São Caetano do Sul (CELAFISCS), Sao Paulo, Brazil; 12 Kenyatta University, Nairobi, Kenya; 13 School of Medicine, Universidad de los Andes, Bogota, Colombia, Colombia; 14 University of Bath, Bath, United Kingdom; 15 Tianjin Women’s and Children’s Health Center, Tianjin, China; The Ohio State University, UNITED STATES

## Abstract

**Purpose:**

Previously, studies examining correlates of sedentary behavior have been limited by small sample size, restricted geographic area, and little socio-cultural variability. Further, few studies have examined correlates of total sedentary time (SED) and screen time (ST) in the same population. This study aimed to investigate correlates of SED and ST in children around the world.

**Methods:**

The sample included 5,844 children (45.6% boys, mean age = 10.4 years) from study sites in Australia, Brazil, Canada, China, Colombia, Finland, India, Kenya, Portugal, South Africa, the United Kingdom, and the United States. Child- and parent-reported behavioral, household, and neighborhood characteristics and directly measured anthropometric and accelerometer data were obtained. Twenty-one potential correlates of SED and ST were examined using multilevel models, adjusting for sex, age, and highest parental education, with school and study site as random effects. Variables that were moderately associated with SED and/or ST in univariate analyses (p<0.10) were included in the final models. Variables that remained significant in the final models (p<0.05) were considered correlates of SED and/or ST.

**Results:**

Children averaged 8.6 hours of daily SED, and 54.2% of children failed to meet ST guidelines. In all study sites, boys reported higher ST, were less likely to meet ST guidelines, and had higher BMI z-scores than girls. In 9 of 12 sites, girls engaged in significantly more SED than boys. Common correlates of higher SED and ST included poor weight status, not meeting physical activity guidelines, and having a TV or a computer in the bedroom.

**Conclusions:**

In this global sample many common correlates of SED and ST were identified, some of which are easily modifiable (e.g., removing TV from the bedroom), and others that may require more intense behavioral interventions (e.g., increasing physical activity). Future work should incorporate these findings into the development of culturally meaningful public health messages.

## Introduction

Physical inactivity and sedentary behavior have been independently associated with a wide range of negative health indicators including obesity, poor cardio-metabolic health, and poor psychosocial health [1–4]. Sedentary behavior is characterized by waking behaviors that require little energy expenditure and that occur in a sitting or reclined position [5]. Total sedentary time (SED) can be further classified by a variety of specific sedentary behaviors such as reading, playing quietly, watching television (TV), using the computer, or playing video games. Previous work has relied largely upon self-reported sedentary pursuits, and focused largely on screen time (ST), which usually entails some combination of watching TV, playing video games, and/or using the computer [4]. Although ST is often used as a proxy measure for SED, ST accounts for only about a third of SED [6], with the rest of the SED being spent in a variety of other pursuits such as reading a book, passive transport, or eating. With the widespread use of accelerometers, researchers are able to capture objectively measured SED throughout the day [7]. This is important since increased ST is consistently associated with poor health in children and youth, but the relationship between increased SED and increased disease risk is less clear [8,9].

In developed countries, those in lower socio-economic levels and those belonging to ethnic minorities are more likely to be sedentary [10,11]. In under-developed, or developing countries, the trend is reversed, with those with the highest socio-economic status being most sedentary [12]. Previous work has also shown that early adolescence is a common time for levels of moderate- to vigorous-intensity physical activity to decline, and for SED (including ST) to increase [10,13]. Further, differences in levels of physical activity and SED due to socio-cultural factors are largely established during this developmental period [10]. Therefore, pre-adolescence may be a particularly important time to prevent the development of poor behavior habits, including decreased participation in physical activity of all intensities, and increased attraction to screen based SED.

A review examining the correlates of TV viewing in youth suggested that those who watched the most TV were pre-adolescents (i.e., aged 9–13 years), from families of lower socio-economic status, from single parent households, and those belonging to ethnic minorities (with African American children watching the most TV) [14]. However, this review was largely informed by North American studies (72% of included studies), and focused exclusively on TV viewing. The authors identify these limitations and go on to recommend that future studies use an ecological framework to understand a broad range of intrapersonal, interpersonal, and environmental correlates of sedentarism. Since this review, many studies have examined correlates of sedentarism, including several that have used direct (i.e., accelerometer) measurement to assess SED; however, they have been limited by small sample sizes, restricted geographic area, and/or little socio-cultural variability [15]. Temmel and Rhodes [15] identified 64 studies that examined the correlates of SED in young people. Although the review included studies from countries all over the world, very few studies included multi-national populations using the same methodology. In fact, the most geographically diverse study sample came from the Health Behaviors in School-aged Children survey (HBSC), which included only self-reported data from developed countries.

The International Study of Childhood Obesity, Lifestyle and the Environment (ISCOLE) represents the most current, directly-measured, harmonized dataset focused on lifestyle and obesity among children from all regions of the world. The aim of the present analysis was to investigate the correlates of SED and ST in a large multi-national sample of 10 year-old children from diverse cultural and socioeconomic backgrounds.

## Methods

### Data Source

ISCOLE is a multi-site, cross-sectional study conducted in 9–11 year-old children from sites in 12 different countries (Australia, Brazil, Canada, China, Colombia, Finland, India, Kenya, Portugal, South Africa, United Kingdom, and United States) [16]. The primary aim of ISCOLE was to determine the relationships between lifestyle behaviors and obesity in a multi-national study of 9–11 year-old children. Further, ISCOLE aimed to investigate the influence of higher-order characteristics such as behavioral settings, and the physical, social and policy environments, on the observed relationships within and between countries [16]. To ensure ISCOLE represented a variety of backgrounds and circumstances, study sites were chosen from diverse geographic regions around the world (i.e., Europe, Africa, the Americas, South-East Asia, and the Western Pacific) and across different levels of socio-economic indicators (i.e., World Bank income classification, Human Development Index, and the Gini Index). Additional details on study design, participating countries, and full methodology have been published elsewhere [16].

Data collection for ISCOLE occurred from October 2011 until December 2013 with a goal of recruiting at least 500 participants, aged 9–11 years, from each study site. Data collection strategies varied slightly by ISCOLE site; details on site-specific recruitment strategies can be found in the ISCOLE methods paper [16]. The ISCOLE coordinating center, located at the Pennington Biomedical Research Center in Baton Rouge, Louisiana, was responsible for overall administration of the study. This project was approved by the relevant research ethics boards at Pennington Biomedical Research Center, at each ISCOLE study site, and at the respective school boards. Written informed parental consent and child assent were obtained for all participants.

### Dependent Variables

#### Accelerometer measured sedentary time

The ActiGraph GT3X+ triaxial accelerometer (ActiGraph LLC, Pensacola, FL, USA) was used to objectively measure SED. Participants were asked to wear the accelerometer, attached to an elastic belt around the waist at the right mid-axillary line, for 7 consecutive days, 24 hours/day, removing only for water activities (e.g., bathing, swimming). Children received instruction during the initial in-school assessment on how to wear the accelerometer. To increase compliance a variety of measures were used across different countries including in-person compliance checks, and phone calls to the participants’ parents to ensure the child was wearing the accelerometer correctly.

Data reduction strategies limited the analytical dataset to participants who provided at least four days of valid measurements (including at least one weekend day), with at least 10 hours/day of waking wear time [17,18]. Data were collected at a sampling rate of 80 Hz, downloaded in 1-second epochs, and aggregated to 15-second epochs for analysis [19]. To determine SED, total sleep period time and non-wear time were identified using validated procedures [20]. For the current analysis, SED was defined as all epochs showing ≤25 counts/15 seconds, consistent with widely used cutoffs from Evenson et al. [19].

#### Self-reported screen time

Child-reported screen time was determined from a Diet and Lifestyle Questionnaire, with questions taken from the U.S. Youth Risk Behaviour Surveillance System [16,21]. Children were asked how many hours they typically watched TV, and how many hours they played video games and/or used the computer during their discretionary time, per week day, and per weekend day. Responses were: 0 = I did not watch TV, 1 = ≤1 hour of TV, 2 = 2 hours, 3 = 3 hours, 4 = 4 hours, 5 = ≥5 hours of TV. A weighted mean score of hours of daily ST was calculated as follows: [(hours of TV on weekdays x 5) + (hours of TV on weekend days x 2) + (hours of video games and computers on weekdays x 5) + (hours of video games and computers on weekend days x 2)]/7. For analysis, this is presented as a ST score, rather than total hours of ST since after 5 hours/day, we could not ascertain the participant’s actual amount of ST. Self-reported methods for quantifying ST have acceptable reliability and validity in children [7]. After testing for normality, ST was log-transformed for analysis and analyzed as a continuous variable. ST was also presented as those who did not meet ST guidelines of ≤2 hours/day [22].

### Potential Correlates

Twenty-one potential correlates of SED and/or ST were included in the analysis. Correlates included directly measured, child-reported, and parent-reported variables and were chosen based on the previous literature and the plausibility of their relationship with SED and/or ST [14,15]. See Table 1 for details on response categories and additional measurement details.

**Table 1:** Potential correlates of objectively measured sedentary time (SED) and self-reported screen time (ST).

Variable	Measurement method	Use in analysis
**Demographic and biological**		
Sex	Parent-report: Demographic and Family History Questionnaire	Binary variable: male, female (used as a covariate)
Percent body fat	Directly measured using Tanita SC-240 Body Composition Analyzer	Continuous
Waist circumference	Directly measured by ISCOLE researcher	Continuous
BMI z-score	Directly measured height and weight, calculated using WHO criteria [24]	Continuous
**Behavioral characteristics**		
Healthy and unhealthy eating pattern scores	Child-reported food frequency questionnaire: ISCOLE Diet and Lifestyle Questionnaire	Continuous: obtained from a principal component analysis derived from a 23-item food frequency questionnaire
Commute to school (main part of journey)	Child-reported: ISCOLE Diet and Lifestyle Questionnaire	Re-coded as dichotomous: active (walking, bicycle/rollerblade/skateboard/scooter), and inactive (bus/train/tram/underground/boat, car/motorcycle/moped)
Sleep (in the past week)	Child-reported: ISCOLE Diet and Lifestyle Questionnaire	Re-coded as dichotomous: “very bad/fairly bad” and “very good/fairly good”
Physical activity	Child-reported: ISCOLE Diet and Lifestyle Questionnaire	Categorical: Child-report engaging in moderate- to vigorous-intensity physical activity for at least 60 minutes (8 responses: 0 days, 1 day, 2 days, 3 days, 4 days, 5 days, 6 days, 7 days). Was included in the model re-coded as those active more and less than 6 days in the past week.
Outdoor time (before school, after school, and weekend)	Child-reported: ISCOLE Diet and Lifestyle Questionnaire	Re-coded as dichotomous: <2 hours and ≥2 hours before school, after school, or on weekends
**Family situation**		
Parental BMI	Parent-reported: Demographic and Family History Questionnaire	Re-coded as dichotomous for mothers and fathers: normal weight, or overweight/obese
Parental work status	Parent-reported: Demographic and Family History Questionnaire	Re-coded as highest level of parental employment: <part time, ≥part time
Parental education	Parent-reported: Demographic and Family History Questionnaire	Re-coded as highest level of parental education (used as a covariate): ≤high school, and >high school
**Home and neighborhood environment**		
Number of TVs in home	Parent-reported: ISCOLE Neighborhood and Home Environment Questionnaire	Re-coded as dichotomous: ≤1, and ≥2
TV in bedroom	Child-reported: ISCOLE Diet and Lifestyle Questionnaire; Parent-reported: ISCOLE Neighborhood and Home Environment Questionnaire	Binary response: yes/no
Computer in the bedroom	Parent-reported: ISCOLE Neighborhood and Home Environment Questionnaire	Binary response: yes/no
Automobile ownership	Parent-reported: ISCOLE Neighborhood and Home Environment Questionnaire	Continuous: number of working automobiles owned per household. Re-coded as dichotomous: <2 and ≥2.
Trusted neighborhood	Parent-reported: ISCOLE Neighborhood and Home Environment Questionnaire	Re-coded as binary: “disagreed/strongly disagreed”, and “agreed/strongly agreed”
High crime in neighborhood	Parent-reported: ISCOLE Neighborhood and Home Environment Questionnaire	Re-coded as dichotomous: “disagreed/strongly disagreed”, and “agreed/strongly agreed”
Neighborhood is walkable	Parent-reported: ISCOLE Neighborhood and Home Environment Questionnaire	Re-coded as dichotomous: “disagreed/strongly disagreed”, and “agreed/strongly agreed”

BMI: body mass index; ISCOLE: International Study of Childhood Obesity, Lifestyle and the Environment; TV: television; WHO: World Health Organization

#### Anthropometric variables

Anthropometric data (i.e., height, weight, waist circumference, percent body fat) were directly measured by trained ISCOLE researchers during an in-school visit according to standardized procedures and measurement tools [16]. Height (to the nearest 0.1 cm) was measured using a Seca 213 portable stadiometer (Hamburg, Germany). Weight (to the nearest 0.1 kg) and body fat percentage (to the nearest 0.1%) were measured using a portable Tanita SC-240 Body Composition Analyzer (Arlington Heights, IL, USA). The Tanita SC-240 has shown acceptable accuracy for estimating body fat percentage when compared with dual-energy X-ray absorptiometry, supporting its use in field studies [23]. Body mass index (BMI) was calculated (kg/m2), and BMI z-score, based on age and sex, was determined using the World Health Organization (WHO) cut-offs for all participants [24]. Waist circumference (to the nearest 0.1 cm) was measured using a non-elastic anthropometric tape after a normal exhalation, at the mid-point between the top of the iliac crest and the bottom of the last floating rib, directly against the skin (except in Australia where measures were taken over light clothing).

#### Child-reported behavioral characteristics

Participants completed the ISCOLE Diet and Lifestyle Questionnaire, containing questions asked to the child related to dietary intake, physical activity, ST, and sleep [16]. The questionnaires were generally completed during the ISCOLE school visit, at the same time that anthropometric measurements were obtained and that the accelerometers were distributed. Children completed a Food Frequency Questionnaire (FFQ) adapted from the HBSC study [25], which asked how often they consumed 23 food items in a usual week. To identify existing dietary patterns among the study population, principal components analyses were carried out using the FFQ food groups as input variables (excluding fruit juices due to low validity) (unpublished analysis). Eigenvalues and a scree plot analysis were used as the criteria for deciding the number of factors extracted. The two criteria lead to similar conclusions and two factors were eventually chosen for each analysis. The factors were then rotated with an orthogonal varimax transformation to force non-correlation of the factors and to enhance the interpretation. Two factors were included in this manuscript as exposure variables: “unhealthy eating pattern score” (e.g., hamburgers, soft drinks, fried food; higher score means worse eating pattern) and “healthy eating pattern score” (e.g., vegetables and fruits; higher score means better eating pattern). Children were asked how they got to school most days (e.g., walking, car), how much time they spent outside (before school, after school, and weekend), and sleep quality and quantity. Finally, to assess if children met current physical activity guidelines, children were asked how many days they engaged in at least 60 minutes of moderate- to vigorous-intensity physical activity [26].

#### Parent-reported home and neighborhood environment

A Demographic and Family History Questionnaire, and a Neighborhood and Home Environment Questionnaire contained questions for parents related to their home and neighborhood environment [16]. The parent filling out the questionnaire was asked information specific for the “mother” and the “father”. In particular, they were asked to report the highest level of education (ranging from “less than high school” to “post graduate degree”), employment (ranging from “not at all” to “full time”), height, and weight for themselves and their co-parent. The parent-report questionnaires were sent home with the child at the same time as the parent-consent forms and collected during the in-school visit. Parents were contacted if there was missing information, or if the responses needed to be clarified.

### Statistical Analysis

Statistical analyses were conducted using SAS 9.4 (SAS Institute Incorporated, North Carolina, USA). Descriptive information (means, standard deviation, frequencies as appropriate) was calculated for demographic and anthropometric characteristics of all participants and their parents. Unpaired t-tests and chi-square tests were used to test differences between boys and girls. Multilevel general linear models (PROC MIXED), including school and ISCOLE study site as random effects, were used to determine correlates of SED and ST. Potential correlates (see Table 1 for details) were first included in univariate models; variables that were at least marginally significant (p<0.10), were subsequently included in domain-specific models similar to those outlined in the social ecological model proposed by Owen et al. for SED [27] (i.e., biological characteristics, behavioral characteristics, parental characteristics, and home and neighborhood environment). Variables that showed at least marginally significant associations (p<0.10) with SED or ST in the domain-specific models were included in the final model (results of domain-specific models not shown). Variables that remained significant (p<0.05) in the final model were considered correlates of SED and/or ST. To show significant correlates across sites, we ran the final models for SED and ST in each site country separately (keeping schools as a random effect). To show differences in levels of SED and ST between boys and girls in each site, we ran sex by site interactions and computed the least square means (LSMEANS) of fixed effects.

Child’s sex, age, and their parent’s highest level of education were included as covariates for all models. These covariates were selected based on the plausibility of their potential confounding effect and because of their known associations with SED and/or ST [15]. The Kenward-Roger approximation (DDFM = KR) was used to calculate degrees of freedom [28]. Multicollinearity was tested using tolerance and variance inflation factors, and results indicated no issues with multicollinearity [29]. Analyses were conducted and presented for the total sample, and separately for boys and girls.

## Results

**Fig 1:**
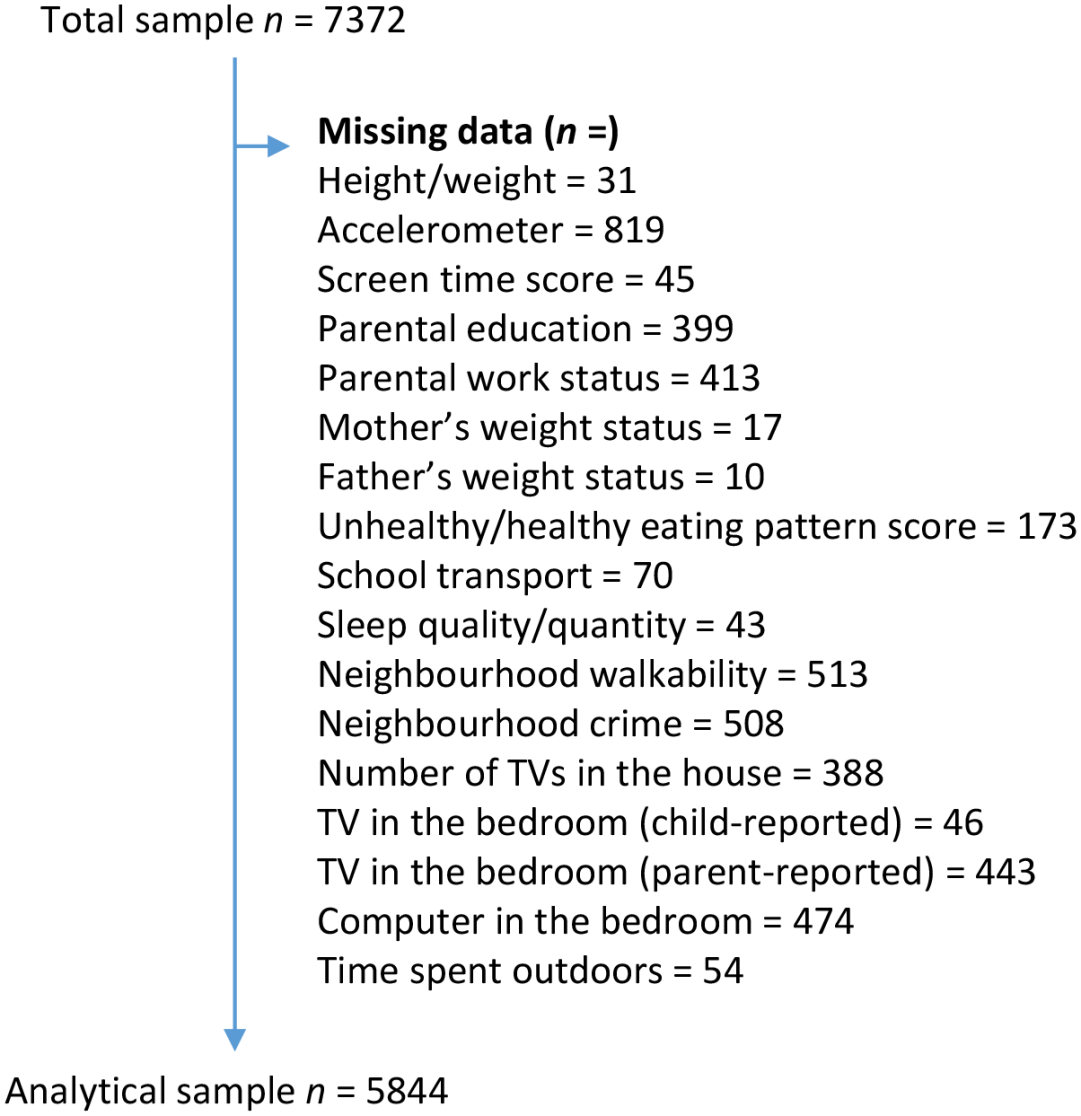
Participants with missing data. A total of 1528 participants were excluded due to missing data, some participants had missing data for more than one variable.

Complete data for the outcomes of interest and all investigated correlates were available for 9–11 year-old children from Australia (n = 454), Brazil (n = 427), Canada (n = 502), China (n = 487), Colombia (n = 836), Finland (n = 542), India (n = 540), Kenya (n = 464), Portugal (n = 547), South Africa (n = 306), United Kingdom (n = 407), and the United States (n = 422). Missing data were found for 1528 participants (Fig 1); due to the large number of participants with missing data, sensitivity analysis was performed (Data not shown). Participants excluded from the present analysis engaged in similar levels of SED, but had higher BMI z-scores, and lower ST scores than those included in the analysis; more boys were also excluded from the analysis due to missing data. As per the ISCOLE study design, included countries varied considerably in their population size and level of wealth and human development. Across the total sample, the largest fraction of the total variance in both SED and ST was explained by individual-level factors (78.0% and 83.7%, respectively), followed by sites (13.0% and 9.2%) and schools (9.1% and 7.1%). Table 2 presents the means and frequencies of the descriptive variables.

**Table 2:** Descriptive characteristics of all participants (*n* = 5,844).

ISCOLE site country (city)	World Bank ranking (income)	Parental higher education^§^ (*n*, (%))	Participants (*n*, (% boys))	Age (years, mean (SD))	Weight Status*(*n*, (%) OW/OB)	SED (hr/day)	ST score^¥^	Not meeting ST guidelines (*n*, (%))
Australia (Adelaide)	High	364 (80.2)	454 (46.0)	10.7 (0.4)	169 (37.2)	7.9 (1.0)	2.8 (1.8)	266 (58.6)
Brazil (Sao Caetano do Sul)	Upper-middle	172 (40.3)	427 (48.0)	10.5 (0.5)	195 (45.7)	8.3 (1.4)	3.7 (2.3)	309 (72.4)
Canada (Ottawa)	High	458 (91.2)	502 (41.6)	10.5 (0.4)^Ɨ^	154 (30.7)^Ɨ^	8.5 (1.0)	2.4 (1.9)	227 (45.2)
China (Tianjin)	Upper-middle	240 (49.3)	487 (52.0)	9.9 (0.5)	204 (41.9)^Ɨ^	9.4 (1.1)	1.9 (1.7)	164 (33.7)
Colombia (Bogotá)	Upper-middle	281 (33.6)	836 (49.3)	10.5 (0.6)^Ɨ^	192 (23.0)^Ɨ^	8.3 (1.1)	2.9 (1.5)	552 (66.0)
Finland (Helsinki, Espoo, Vantaa)	High	331 (73.2)	452 (46.9)	10.5 (0.4)	110 (24.3)	8.8 (1.2)	2.7 (1.7)	257 (56.9)
India (Bangalore)	Lower-middle	448 (83.0)	540 (45.6)	10.4 (0.5)	173 (32.0)	8.6 (1.1)	1.8 (1.3)	169 (31.3)
Kenya (Nairobi)	Low	298 (64.2)	464 (45.9)	10.2 (0.7)	90 (19.4)	8.2 (1.1)	2.4 (1.7)	246 (53.0)
Portugal (Porto)	High	116 (21.1)	547 (43.0)	10.4 (0.3)	250 (45.7)^Ɨ^	9.2 (1.0)	2.3 (1.5)	265 (48.5)
South Africa (Cape Town)	Upper-middle	91 (29.7)	306 (40.0)	10.2 (0.7)	80 (26.1)	8.2 (1.1)	3.1 (2.1)	191 (62.4)
UK (Bath, North East Somerset)	High	294 (72.2)	407 (42.8)	10.9 (0.5)	111 (27.3)	8.3 (1.0)	2.9 (1.7)	275 (67.6)
US (Baton Rouge)	High	313 (74.2)	422 (41.0)	9.9 (0.6)^Ɨ^	160 (37.9)	8.7 (1.0)	3.1 (2.3)	247 (58.5)
**All sites**		**3406 (58.3)**	**5844 (45.6)**	**10.4 (0.6)**	**1888 (32.3)** ^Ɨ^	**8.6 (1.2)**	**2.6 (1.8)**	**3158 (54.2)**

**^§^**Number (%) of sample who had at least one parent complete more than at least high school education (i.e., at least some college/university).

*Number (%) with WHO BMI z-score classification overweight or obese

^Ɨ^Sites where boys had significantly higher values than girls (p<0.05).

^¥^ST score = [(hours of TV on weekdays x 5) + (hours of TV on weekend days x 2) + (hours of video games and computers on weekdays x 5) + (hours of video games and computers on weekend days x 2)]/7

^ǂ^Number (%) of children not meeting guidelines for ≤2 hours of screen time/day, in all sites, girls were significantly more likely to meet guidelines than boys (p<0.05).

BMI: Body Mass Index; SED: sedentary time; SD: standard deviation; ST: screen time; UK: United Kingdom; US: United States; OW/OB: overweight/obese.

**Fig 2:**
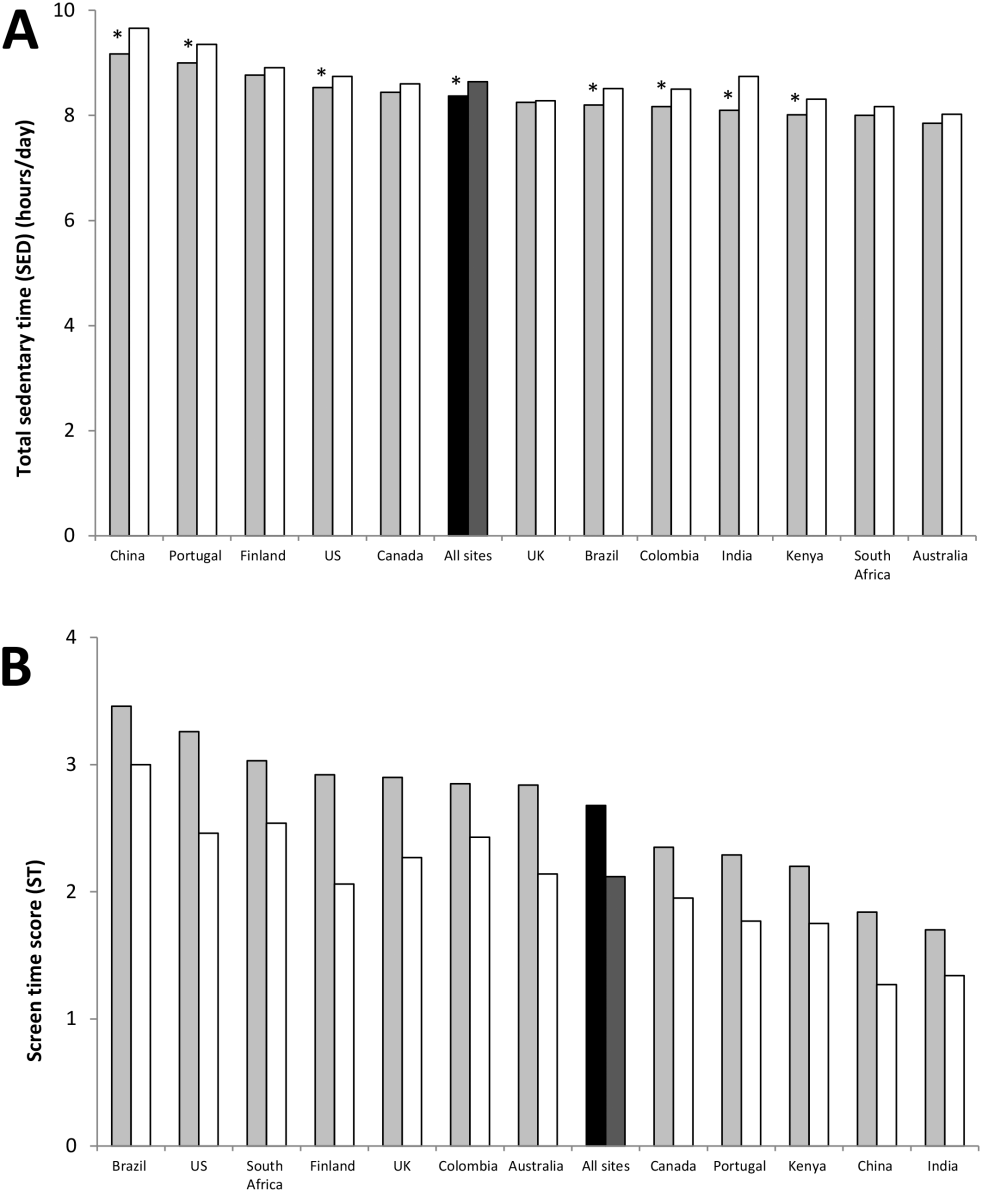
Mean total sedentary time (SED) and screen time (ST) score. Mean accelerometer measured SED (Panel A) and self-reported ST (Panel B) for boys (light grey bars), and girls (white bars). Black bars (boys) and dark grey bars (girls) represent overall sample means. Panel A: Accelerometer measured total sedentary time (SED) (hours/day) across all 12 ISCOLE sites (*indicates sites where girls engaged in significantly more SED than boys, p<0.05). Panel B: Self-reported screen time (ST) score across all 12 ISCOLE country sites (in all sites boys had significantly higher values for ST than girls, p<0.05).

Fig 2 shows the mean SED (Panel A), and ST scores (Panel B) across all ISCOLE sites. Overall, boys had higher ST scores (mean difference = 0.57), were less likely to meet ST guidelines (54.2% of boys versus 68.2% of girls), and tended to have higher BMI z-scores (mean difference = 0.16) than girls. In several study sites (9/12), girls engaged in significantly more SED than boys (mean difference = 0.29 hours/day). China, Portugal, Finland, the US, and Canada had higher levels of SED than the ISCOLE average (8.6 hours/day). Brazil, the US, South Africa, Finland, the UK, Colombia, and Australia had higher ST scores than the ISCOLE average (2.6 hours/day). India had the highest percent (68.7%), and Brazil had the lowest percent (27.6%) of participants meeting ST guidelines (≤2 hours per day) [22].

### Univariate Analyses

The results of the univariate regression analyses are presented in Tables 3 and 4. Of the 21 potential biological, behavioral, parental, home, and neighborhood correlates, 13 were significantly associated with SED in the total sample (14 for boys, and 12 for girls) and 17 were significantly associated with ST in the total sample (16 for boys, and 18 for girls).

**Table 3:** Univariate correlates of total sedentary time (SED) (*n* = 5,844)^a^.

	Total			Boys			Girls		
Variables	β-coefficient	SE	*p*-value	β-coefficient	SE	*p*-value	β-coefficient	SE	*p*-value
**Biological**									
Percent body fat^b^	**0.83**	**0.11**	**<0.0001**	**1.10**	**0.18**	**<0.0001**	**0.71**	**0.15**	**<0.0001**
Waist circumference	**0.63**	**0.09**	**<0.0001**	**0.76**	**0.14**	**<0.0001**	**0.55**	**0.13**	**<0.0001**
BMI z-score^c^	**2.71**	**0.67**	**<0.0001**	**3.02**	**1.00**	**0.0026**	**2.86**	**0.90**	**0.0014**
**Behavioral**									
Unhealthy eating pattern score	**-6.50**	**1.01**	**<0.0001**	**-6.37**	**1.46**	**<0.0001**	**-6.35**	**1.42**	**<0.0001**
Healthy eating pattern score	**-2.81**	**0.86**	**0.0011**	**-2.92**	**1.33**	**0.0282**	**-2.58**	**1.14**	**0.0242**
Passive commute to school	**-3.62**	**2.02**	**0.0744**	**-6.59**	**3.01**	**0.0289**	-2.25	2.67	0.3991
Poor sleep quality	4.88	3.06	0.1108	**8.07**	**4.62**	**0.0809**	1.95	4.13	0.6377
Poor sleep quantity	2.85	2.77	0.3024	**6.81**	**4.05**	**0.0933**	0.21	3.84	0.9561
Not meeting PA guidelines	**10.33**	**2.07**	**<0.0001**	**14.89**	**3.05**	**<0.0001**	**5.63**	**2.83**	**0.0472**
<2 hours outside before school	**11.31**	**3.13**	**0.0003**	**17.61**	**4.74**	**0.0002**	6.61	4.16	0.1120
<2 hours outside after school	**13.44**	**1.73**	**<0.0001**	**17.13**	**2.66**	**<0.0001**	**10.28**	**2.268**	**<0.0001**
<2 hours outside weekend	**7.47**	**1.89**	**<0.0001**	**9.99**	**3.04**	**0.0010**	**5.67**	**2.41**	**0.0185**
**Parental characteristics**									
Mother’s weight status	1.71	1.69	0.3110	0.99	2.62	0.7083	1.80	2.21	0.4167
Father’s weight status	**3.28**	**1.66**	**0.0471**	2.57	2.57	0.3170	**3.95**	**2.16**	**0.0679**
Parental work	1.33	1.70	0.4312	1.69	2.72	0.5354	0.39	2.27	0.8639
**Home and neighborhood**									
Number of TVs in home	0.53	2.00	0.7872	0.36	3.06	0.9060	-0.66	2.56	0.7963
TV in bedroom (child-report)	**3.93**	**1.89**	**0.0372**	2.34	2.87	0.4139	**4.64**	**2.50**	**0.0636**
TV in bedroom (parent-report)	1.83	1.89	0.3360	**7.24**	**2.88**	**0.0119**	-2.62	2.51	0.2964
Computer in bedroom (parent-report)	**9.17**	**1.94**	**<0.0001**	**13.64**	**2.98**	**<0.0001**	**6.07**	**2.56**	**0.0174**
Automobile ownership	-4.07	2.81	0.1469	6.52	4.33	0.1321	**-12.97**	**3.68**	**0.0004**
Trusted neighborhood	-1.70	2.20	0.4399	-2.94	3.38	0.3842	-1.03	2.93	0.7243
High crime neighborhood	2.15	2.00	0.2798	0.64	3.14	0.8384	3.11	2.54	0.2218
Walkable neighborhood	-0.95	1.75	0.5863	1.07	2.72	0.6932	-2.84	2.28	0.2120

^a^Multi-level general linear model controlling for sex, age, and highest parental education, with school and ISCOLE site as a random effects; unstandardized beta coefficients and standard errors are presented.

^b^ISCOLE used a variety of measures to assess adiposity, all of which were significant in univariate analyses. Akaike information criterion, Bayesian information criterion, and level of significance were used to determine which measure of adiposity provided the closest fit for the data. Percent body fat alone provided the best fit.

^c^WHO BMI z-score classification. PA: physical activity; SE: Standard error; TV: television

**Table 4:** Univariate correlates of total screen time (ST) (*n* = 5,844)^a^.

	Total			Boys			Girls		
Variables	β-coefficient	SE	*p*-value	β-coefficient	SE	*p*-value	β-coefficient	SE	*p*-value
**Biological**									
Percent body fat	**0.004**	**0.001**	**<0.0001**	**0.004**	**0.001**	**0.0013**	**0.004**	**0.001**	**0.0001**
Waist circumference^b^	**0.003**	**0.001**	**<0.0001**	**0.003**	**0.001**	**0.0022**	**0.004**	**0.001**	**<0.0001**
BMI z-score^c^	**0.02**	**0.01**	**<0.0001**	**0.03**	**0.01**	**0.0002**	**0.02**	**0.01**	**0.0002**
**Behavioral**									
Unhealthy eating pattern score	**0.14**	**0.01**	**<0.0001**	**0.14**	**0.01**	**<0.0001**	**0.15**	**0.01**	**<0.0001**
Healthy eating pattern score	**-0.06**	**0.01**	**<0.0001**	**-0.05**	**0.01**	**<0.0001**	**-0.07**	**0.01**	**<0.0001**
Passive commute to school	0.02	0.02	0.2468	0.00	0.02	0.8717	**0.04**	**0.02**	**0.0463**
Poor sleep quality	**0.09**	**0.02**	**<0.0001**	**0.07**	**0.03**	**0.0352**	**0.11**	**0.03**	**0.0008**
Poor sleep quantity	**0.08**	**0.02**	**<0.0001**	**0.08**	**0.03**	**0.0072**	**0.09**	**0.03**	**0.0032**
Not meeting PA guidelines	**0.04**	**0.02**	**0.0173**	0.03	0.02	0.1599	**0.05**	**0.02**	**0.0368**
<2 hours outside before school	**-0.02**	**0.02**	**<0.0001**	**-0.21**	**0.03**	**<0.0001**	**-0.19**	**0.03**	**<0.0001**
<2 hours outside after school	**-0.08**	**0.01**	**<0.0001**	**-0.07**	**0.02**	**<0.0001**	**-0.08**	**0.02**	**<0.0001**
<2 hours outside weekend	**-0.10**	**0.01**	**<0.0001**	**-0.11**	**0.02**	**<0.0001**	**-0.10**	**0.20**	**<0.0001**
**Parental characteristics**									
Mother’s weight status	**0.03**	**0.01**	**0.0071**	**0.07**	**0.02**	**<0.0001**	0.01	0.02	0.5919
Father’s weight status	0.01	0.01	0.5097	0.03	0.02	0.1500	-0.01	0.02	0.5696
Parental work	0.00	0.01	0.9713	0.01	0.02	0.5257	-0.01	0.02	0.7826
**Home and neighborhood**									
Number of TVs in home	**-0.07**	**0.01**	**<0.0001**	**-0.08**	**0.02**	**0.0004**	**-0.08**	**0.02**	**<0.0001**
TV in bedroom (child-report)	**0.16**	**0.01**	**<0.0001**	**0.20**	**0.02**	**<0.0001**	**0.13**	**0.02**	**<0.0001**
TV in bedroom (parent-report)	**0.11**	**0.01**	**<0.0001**	**0.14**	**0.02**	**<0.0001**	**0.10**	**0.02**	**<0.0001**
Computer in bedroom (parent-report)	**0.07**	**0.01**	**<0.0001**	**0.09**	**0.02**	**<0.0001**	**0.05**	**0.02**	**0.0130**
Automobile ownership	**0.06**	**0.02**	**0.0045**	**0.06**	**0.03**	**0.0688**	**0.06**	**0.03**	**0.0434**
Trusted neighborhood	0.01	0.02	0.7502	0.02	0.02	0.4567	0.04	0.02	0.8572
High crime neighborhood	0.00	0.01	0.8595	0.00	0.02	0.8237	-0.01	0.02	0.3221
Walkable neighborhood	-0.02	0.01	0.1416	-0.02	0.02	0.3283	-0.02	0.01	**<0.0001**

^a^Multi-level general linear model controlling for sex, age, and highest parental education, with school and ISCOLE site as a random effects; unstandardized beta coefficients and standard errors are presented.

^b^ISCOLE used a variety of measures to assess adiposity, all of which were significant in univariate analyses. Akaike information criterion, Bayesian information criterion, and level of significance were used to determine which measure of adiposity provided the closest fit for the data. Waist circumference alone provided the best fit.

^c^WHO BMI z-score classification. PA: physical activity; SE: Standard error; TV: television

### Multivariable Analyses

Results of the final multivariable regression models are presented in Table 5. In the final models, 7 correlates of SED (8 in boys and 5 in girls) and 10 correlates of ST (10 in boys and 10 in girls) were identified. This included four common correlates across the total sample: adiposity (percent body fat for SED and waist circumference for ST), TV in the bedroom (child-reported), computer in the bedroom, and meeting physical activity guidelines. To show significant correlates across sites, we ran the final models for SED and ST in each site country separately (keeping school as a random effect). Table 6 shows significant correlates (and their direction) in each ISCOLE site. For total SED, correlates that were most common across sites were percent body fat (positive association), and unhealthy eating pattern score (negative association). For ST, correlates that were most common across sites were healthy (negative association) and unhealthy (positive association) eating pattern scores.

**Table 5:** Final model for correlates of accelerometer measured total sedentary time (SED) and self-reported screen time (ST) (*n* = 5,844).

	Total			Boys			Girls		
Variables	β-coefficient	SE	*p*-value	β-coefficient	SE	*p*-value	β-coefficient	SE	*p*-value
**Final model for SED** ^a^									
Percent body fat	**0.76**	**0.11**	**<0.0001**	**0.98**	**0.18**	**<0.0001**	**0.68**	**0.15**	**<0.0001**
Father’s weight status	-1.49	1.66	0.3702	0.38	2.55	0.8802	-2.70	2.18	0.2168
TV in bedroom (child-report)	**-7.10**	**2.40**	**0.0031**	**-9.75**	**3.58**	**0.0065**	-4.18	3.24	0.1967
TV in bedroom (parent-report)	**5.12**	**2.41**	**0.0337**	**10.11**	**3.60**	**0.0050**	-0.22	3.25	0.9468
Computer (parent-report)	**8.57**	**1.96**	**<0.0001**	**11.99**	**3.00**	**<0.0001**	**6.30**	**2.59**	**0.0150**
Car ownership	-3.15	2.79	0.2592	7.13	4.26	0.0943	**-12.37**	**3.68**	**0.0008**
Unhealthy eating pattern Score	**-5.05**	**1.03**	**<0.0001**	**-4.57**	**1.47**	**0.0019**	**-5.03**	**1.44**	**0.0005**
Not meeting PA guidelines	**8.01**	**3.16**	**0.0001**	**11.52**	**3.06**	**0.0002**	3.95	2.86	0.1679
<2 hours outside before school	5.76	3.16	0.0683	**12.54**	**4.77**	**0.0085**	1.20	4.21	0.7748
<2 hours outside after school	**10.81**	**1.76**	**<0.0001**	**12.99**	**2.70**	**<0.0001**	**8.83**	**2.33**	**0.0001**
**Final model for ST**									
Waist circumference	**0.003**	**0.001**	**<0.0001**	**0.003**	**0.001**	**0.0020**	**0.004**	**0.001**	**<0.0001**
Mother weight status	0.02	0.01	0.1104	0.05	0.02	0.0053	-0.00	0.02	0.9279
Number of TVs in the home	**-0.03**	**0.01**	**0.0317**	-0.02	0.02	0.2553	**-0.05**	**0.02**	**0.0167**
TV in bedroom (child-report)	**0.10**	**0.01**	**<0.0001**	**0.13**	**0.02**	**<0.0001**	**0.07**	**0.02**	**0.0001**
Computer (parent-report)	**0.04**	**0.01**	**0.0022**	**0.06**	**0.02**	**0.0039**	0.03	0.02	0.1102
Car ownership	0.02	0.02	0.2279	0.02	0.03	0.4503	0.02	0.03	0.4354
Unhealthy eating pattern score	**0.13**	**0.01**	**<0.0001**	**0.12**	**0.01**	**<0.0001**	**0.14**	**0.01**	**<0.0001**
Healthy eating pattern score	**-0.07**	**0.01**	**<0.0001**	**-0.06**	**0.01**	**<0.0001**	**-0.07**	**0.01**	**<0.0001**
Not meeting PA guidelines	**0.04**	**0.01**	**0.0044**	0.03	0.02	0.1047	**0.05**	**0.02**	**0.0111**
<2 hours outside before school	**-0.15**	**0.02**	**<0.0001**	**-0.14**	**0.03**	**<0.0001**	**-0.15**	**0.03**	**<0.0001**
<2 hours outside after school	**-0.04**	**0.01**	**0.0012**	**-0.04**	**0.02**	**0.0308**	**-0.04**	**0.02**	**0.0181**
<2 hours outside on weekends	**-0.09**	**0.01**	**<0.0001**	**-0.09**	**0.02**	**<0.0001**	**-0.08**	**0.02**	**<0.0001**

^a^Multi-level general linear model controlling for sex, age, and highest parental education, with school and ISCOLE site as a random effects; unstandardized beta coefficients and standard errors are presented. PA: physical activity; SE: Standard Error; SED: accelerometer measured sedentary time; ST: self-reported screen time; TV: television

**Table 6:** Significant correlates by ISCOLE country site for total sedentary time (SED) and screen time (ST).

	Australia	Brazil	Canada	China	Colombia	Finland	India	Kenya	Portugal	South Africa	U.K.	U.S.
**Final model for SED** ^a^												
Percent body fat	+		+		+	+					+	+
Father weight status		−										
TV (child-report)								+		−		
TV (parent-report)				−						+		
Computer (parent-report)				+		+					+	
Car ownership				−								
Unhealthy eating pattern score		−	−	−	−							
Not meeting PA guidelines	+					+						+
<2 hours outside before school						+						
<2 hours outside after school	+	+			+	+			+	+	+	+
**Final model for ST^b^**												
Waist circumference	+		+		+	+	+				+	
Mother weight status	−											
Number of TVs in the home			−				+					
TV (child-report)				+					+		+	+
Computer (parent-report)		+	+						+			
Car ownership												
Unhealthy eating pattern score	+	+	+	+	+	+	+	+	+	+	+	+
Healthy eating pattern score	−		−	−	−	−	−		−			−
Not meeting PA guidelines							+					
<2 hours outside before school			−				−	−		−		
<2 hours outside after school				−						−		
<2 hours outside on weekends				−			−	−	−			

^a^Multi-level general linear model controlling for sex, age, and highest parental education, with school as a random effect. (+) indicates a direct relationship between SED or ST and the associated correlate; (−) indicates an inverse relationship between SED or ST and the associated correlate. A blank cell indicates no significant association was found for the particular correlate in the respective country. PA: physical activity; SED: accelerometer measured sedentary time; ST: self-reported screen time; TV: television; U.K.: United Kingdom; U.S.: United States.

#### Total sedentary time

Across the total sample, SED was positively associated with percent body fat, not meeting physical activity guidelines, time outside after school, TV in the bedroom (parent-reported), and computer in the bedroom. SED was negatively associated with unhealthy eating pattern score, and TV in the bedroom (child-reported). In other words, children who were *more* sedentary had higher body fat, were less active, spent less time outside after school, and had a computer, and a TV in their bedroom (via parental report). Children who were *less* sedentary ate *more* unhealthy food, and self-reported that they *didn’t* have a TV in their bedroom.

For boys, SED was positively associated with percent body fat, not meeting physical activity guidelines, time outside before, or after school, TV in the bedroom (parent-reported), and computer in the bedroom; SED was negatively associated with unhealthy eating pattern score, and TV in the bedroom (child-reported). That is to say, when looking at just boys, those who were *more* sedentary, had higher body fat, were less active, spent less time outside, and had a computer and a TV in their bedroom (via parental report). Boys who were *less* sedentary ate *more* unhealthy food, and self-reported that they *didn’t* have a TV in their bedroom.

For girls, SED was positively associated with percent body fat, computer in the bedroom, and spending less than 2 hours outside after school; SED was negatively associated with household car ownership, and unhealthy eating pattern score. So, when just looking at girls, those who were *more* sedentary had higher body fat, spent less time outside, and had a computer in their bedroom. Girls who were *less* sedentary ate *more* unhealthy food, and had higher household car ownership.

#### Screen time

Across the total sample, ST was positively associated with waist circumference, TV in the bedroom (child-reported), computer in the bedroom, unhealthy eating pattern score, and meeting physical activity guidelines. ST was negatively associated with number of TVs in the house, healthy eating pattern score, and spending <2 hours/day outside before school, after school, or on weekends. In other words, for the whole sample, children who reported *more* ST were less active, ate more junk food, and were more likely to have a TV and a computer in their bedroom. Children who reported *less* ST had a healthier diet, spent less time outside, and had more TVs in their house.

For ST, correlates for boys and girls alone were the same as those of the total sample, with a few exceptions. Correlates for boys were the same as those identified for the total sample except we saw no significant association with ST and number of TVs in the house. For girls, correlates of ST also included household car ownership, and did not include having a computer in their bedroom.

For analysis of SED, potential correlates (except weight status and healthy/unhealthy eating pattern scores) were re-coded as dichotomous variables. The advantage of this analytical approach was that all variables were on approximately the same scale, so that the strength of the association of each correlate could be examined, and interpreted as a proportional increase in the outcome variable (e.g., a unit increase in the beta coefficient for a correlate of SED represented an increase of one minute/day of SED). This information can provide guidance as to which correlates should be targeted in future work. The correlates that showed the strongest association with higher SED were time outside after school (≥2 hours), having a computer in the bedroom (parent-report), and not meeting physical activity guidelines (child-report). These variables represented approximately 10.8 minutes, 8.6 minutes, and 8.0 minutes of SED, respectively. Because ST was measured as a score, instead of hours/day, it could not be interpreted the same way.

## Discussion

This study aimed to identify biological, behavioral, parental, home, and neighborhood correlates of SED, and ST in 10 year-old children from study sites in 12 countries around the world. To date, this represents the most geographically and culturally diverse study sample, and is one of few studies to examine both objectively measured SED, and self-reported ST in the same population. We were able to identify four common correlates of SED and ST (adiposity, TV in the bedroom, computer in the bedroom, and time outside after school). Overall, we were able to identify a greater number of correlates for ST than for SED, and many of the correlates we identified were the same among boys and girls.

Many correlates identified here are consistent with previous work [15]. Previous work has consistently shown that boys engage in more ST, and are less likely to meet ST guidelines than girls [15,30–32], while girls accumulate more SED than boys [15,33]. Many studies have also linked higher levels of sedentarism with greater body weight or adiposity, and with availability of media equipment in the home [15]. It is interesting that we found different results for TV in the bedroom depending on whether the question was answered by the child, or the parent. Previous work has shown that having a TV in the bedroom is associated with higher ST [15], greater risk for obesity [34], higher cardiometabolic risk [35], lower physical activity [36], and shorter self-reported sleep [37]. A possible explanation is social-desirability bias of either the children (i.e., who want to show-off), or the parents (i.e., who know that TV in the bedroom is bad). It is also possible that there was some misunderstanding since many people use a computer or a tablet to watch TV programming, and did or did not count a computer screen as a having TV in the bedroom. This may also help to explain why having a computer in the bedroom was associated with such a large effect on SED (approximately 8.6 minutes/day more than not having a TV in the bedroom). In previous work, Roemmich et al., reported that greater ST was associated with the number of TVs in the home for girls, but not boys [38]. This is consistent with our results; however, we saw that having more TVs in the home was negatively associated with ST score. Possible explanations for this include the placement of the TVs (e.g., the TV could be in the parent’s bedroom, and not accessible for the child), or parental rules regarding TV usage (e.g., one hour per evening).

Consistent with previous work, we saw that greater outdoor time was associated with lower SED [39]; however, we also saw that greater outdoor time (before school, after school, and on weekends) was associated with a higher ST score. To explain this relationship, we ran the final model for ST score, adjusting for accelerometer measured moderate- to vigorous-intensity physical activity, and then again adjusting for SED, but the association remained significant in both instances. It is possible that children who spent more time outside were compensating with higher ST when they were inside. This warrants further exploration. Another perplexing finding was the relationship with SED and unhealthy eating pattern score. One would assume that higher unhealthy eating pattern scores would be associated with higher levels of SED and ST (e.g., those who were more sedentary, or watched more TV, ate more unhealthy food). We found that this relationship was true for the relationship between ST score and unhealthy eating pattern score; however, our analysis showed that *lower* SED was associated with *higher* unhealthy eating pattern score (i.e., children who were *less* sedentary ate more junk food). The reason for this is not obvious and warrants further exploration.

Throughout this paper, we used self-reported physical activity instead of using accelerometer derived moderate- to vigorous-intensity physical activity. This is because there is debate as to the appropriateness of including more than one movement variable in the same model. Although the movement variables may not be highly correlated (i.e., no issues with multicollinearity), they are still dependent on each other since they are derived from the same measurement device (i.e. proportional error). Including accelerometer-derived moderate- to vigorous-intensity physical activity variables can also mask variance that could be attributed to other potential correlates. We did examine this to determine if changed the significance of our correlates. For SED, the correlates did not change, with the addition of household car ownership that became significant (p = 0.03). For ST, all correlates remained the same.

To examine how correlates might differ across ISCOLE sites, and to help inform public health strategies and messaging, final models were examined for each country. Overall, when significant, the direction of the association was consistent across sites. This is especially interesting given the large geographical, socio-economical, and culturally diverse sample. Percent body fat and time outside after school were the most common correlates of SED, and eating pattern scores (healthy and unhealthy) were the most common correlates of ST. This information can be used to provide harmonized, as well as country-specific, public health messages in different countries.

Until recently, sedentary behavior research has relied on self-reported measures, and has primarily examined screen-based behaviors. With the widespread use of accelerometers, we are now able to capture SED throughout the day; however, high levels of SED are not always a good predictor of high ST. For example, in ISCOLE, participants from the China site engaged in the highest amount of SED (9.4 hours/day), but had the second lowest ST score (approximately 1.9 hours/day). Similarly, boys reported more ST than girls across all sites, but in the majority of sites, girls engaged in more SED. Across the whole sample, self-reported ST score explained only a small portion (approximately 30%) of accelerometer measured SED. This is consistent with previous work that suggests that although ST is an important aspect of sedentary time, it may not be a good marker for SED [6], and highlights the idea that researchers should be examining a variety of sedentary pursuits.

While accelerometers are able to provide data related to movement/non-movement patterns for the whole day, they are not able to discriminate between different postures (e.g., standing still versus sitting still), or types of sedentary behaviors (e.g., reading versus watching TV) [40]. This suggests that there is a large proportion of daily SED that is unaccounted for by questioning on ST alone. Results from this work suggest that current sedentary behavior guidelines for children and youth that recommend minimizing all SED [22] could be clarified by focusing specifically on reducing ST, with future work aimed at understanding related health effects (both negative *and* positive) of other, non-screen based sedentary behaviors (e.g., reading a book, coloring). Future research should also try and disentangle sedentary multi-tasking (e.g., watching TV, while texting on a smartphone, and surfing the internet) and the related health effects.

Finally, it is important to note that although our findings are supported by previous work, with the emergence of sedentary behavior research, it is possible that not all studies have used the same definitions, or cut-points, for SED or ST that we have used here. The difference in measurement procedures, cut-points used or definitions of SED and/or ST may explain some of the differences in results that have been presented.

### Strengths and Limitations

The work presented here was restricted to children 9–11 years of age and therefore limits the generalizability to other age groups. However, while TV viewing and other screen-based sedentary behaviors are expected to change with age, it has been suggested that there is not sufficient rationale to assume correlates of SED and ST vary also [14]. ISCOLE also relied on child-reported ST; it would have been beneficial to ask both the parents and the children to report on time spent engaging in ST to understand their unique perspectives. Parental report could also provide additional information on parent-related behaviors (e.g., parental ST habits, household rules for ST), giving additional insight into the home environment. ST in ISCOLE is also limited to recreational screen-based activities and does not examine other sedentary behaviors such as reading or drawing (for pleasure or for school). This could be ameliorated by using time-use surveys; however, time-use surveys require additional time to administer and are not feasible for population-based studies [7]. Finally, this work relied largely on child-report questionnaires, which don’t necessarily provide an accurate reflection of the true situation.

The major strengths of ISCOLE are associated with the overall study design and administration [16]. ISCOLE is the most culturally and geographically diverse, up-to-date, and robust study on lifestyles associated with obesity-related health in children. The ISCOLE framework and coordinating center ensured all study sites, and all ISCOLE researchers, completed mandatory training for all aspects of the study. ISCOLE also made use of direct measurement for all anthropometric variables, and accelerometers for all activity variables. Accelerometers have been shown to be a valid tool to measure movement at all levels of intensity, and the cut-points used in this analysis have been shown to have a high sensitivity for physical activity and for SED [19]. It is possible that the use of inclinometers, rather than traditional accelerometers may better capture time spent in SED, but they are more obtrusive to attach to the participant and generally considered uncomfortable to wear for long time periods [7].

### Conclusion

Many common correlates of SED and ST were identified in this large, global, and culturally and socioeconomically diverse sample. Some of these are easily modifiable (e.g., removing a TV or a computer from the bedroom), and others may require more intense behavioral interventions (e.g., increasing habitual physical activity to meet current guidelines). The results of this study support the idea that a single strategy to reduce SED and ST is unlikely to be effective across many countries; however, a strategy aimed at similar behaviours (i.e., correlates identified here), with country-specific interventions, is possible. Future work should adapt these findings to provide culturally meaningful public health strategies and messages and test these correlates in a multi-faceted intervention to reduce SED and ST in children around the world. This may help to improve lifestyle behaviors such as physical activity, reduce excessive time spent in SED and ST, and ultimately reduce the risk of preventable chronic diseases such as obesity worldwide.
